# What Matters to Us: Impact of Telemedicine During the Pandemic in the Care of Patients With Sarcoma Across Scotland

**DOI:** 10.1200/GO.20.00599

**Published:** 2021-06-30

**Authors:** Holly M. McCabe, Alannah Smrke, Fiona Cowie, Jeff White, Peter Chong, Steven Lo, Ashish Mahendra, Sanjay Gupta, Michelle Ferguson, David Boddie, Walter Mmekka, Lorraine Stirling, Lindsay Campbell, Robin L. Jones, Ioanna Nixon

**Affiliations:** ^1^ Department of Management Science, University of Strathclyde, Glasgow, United Kingdom; ^2^ The Royal Marsden Hospital NHS Foundation Trust, London, United Kingdom; ^3^ Scottish Sarcoma Network, The Beatson West of Scotland Cancer Centre, Glasgow, United Kingdom; ^4^ Scottish Sarcoma Network, Gartnavel General Hospital, Glasgow, United Kingdom; ^5^ Scottish Sarcoma Network, Glasgow Royal Infirmary, Glasgow, United Kingdom; ^6^ Scottish Sarcoma Network, Ninewells Hospital, Dundee, United Kingdom; ^7^ Scottish Sarcoma Network, Aberdeen Royal Infirmary, Aberdeen, United Kingdom; ^8^ Scottish Sarcoma Network, Raigmore Hospital, Inverness, United Kingdom; ^9^ The Institute of Cancer Research, London, United Kingdom

## Abstract

In Scotland, approximately 350 sarcoma cases are diagnosed per year and treated in one of the five specialist centers. Many patients are required to travel long distances to access specialist care. The COVID-19 pandemic brought a number of rapid changes into the care for patients with cancer, with increasing utilization of telemedicine. We aimed to evaluate how the utilization of telemedicine affects professionals and patients across Scotland and care delivery, at the Beatson West of Scotland Cancer Centre Sarcoma Unit.

**METHODS:**

Between June 8 and August 25, 2020, we invited patients and professional sarcoma multidisciplinary team members to participate in separate online anonymous survey questionnaires, to assess their attitudes toward telemedicine. Data were extracted, and descriptive statistics were performed.

**RESULTS:**

Patient satisfaction (n = 64) with telemedicine was high (mean = 9.4/10) and comparable with traditional face-to-face appointments (mean = 9.5/10). Patients were receptive to the use of telemedicine in certain situations, with patients strongly opposed to being told bad news via telemedicine (88%). Providers recommended the use of telemedicine in certain patient populations and reported largely equivalent workloads when compared with traditional consultations. Providers reported that telemedicine should be integrated into regular practice (66%), with patients echoing this indicating a preference for a majority of telemedicine appointments (57%).

**CONCLUSION:**

Telemedicine in sarcoma care is favorable from both clinician and patient perspectives. Utilization of telemedicine for patients with rare cancers such as sarcomas is an innovative approach to the delivery of care, especially considering the time and financial pressures on patients who often live a distance away from specialist centers. Patients and providers are keen to move toward a more flexible, mixed system of care.

## INTRODUCTION

The current COVID-19 global pandemic, caused by the novel beta-coronavirus Severe Acute Respiratory Syndrome coronavirus 2, has spurred on the implementation of containment measures such as quarantine, self-isolation, and physical distancing across the country, including in healthcare settings, in an attempt to stem the spread.^1,2^ Patients with cancer have been cited as being at higher risk of serious infection, seen in an outbreak at a cancer center in Edinburgh, Scotland, which resulted in multiple deaths.^3,4^ Patients with cancer may not only be more susceptible to COVID-19, but also incur greater complications as a result of being immunocompromised because of malignancy or treatment regimes, pulmonary disease, and more frequent exposure to the virus via the healthcare system.^5^ To mitigate risks as far as reasonably possible, it was deemed necessary to restructure how care is delivered to patients with cancer.

**CONTEXT**

**Key Objective**
In Scotland, the use of telemedicine in the delivery of cancer care was implemented in a time-pressured environment, in response to the COVID-19 pandemic. It had not been used at this scale in the care of patients with sarcoma previously in Scotland. So, the question becomes what are the implications of telemedicine for patients and practitioners in the area of rare cancers? Therefore, we explore the attitudes of both patients and practitioners toward telemedicine and the implications for the future care of patients with rare cancers such as sarcomas.
**Knowledge Generated**
In our sample, patients and practitioners were widely receptive to the use of telemedicine, indicating that it should become part of regular. Barriers to efficiency were identified, including lack of ability to perform a physical examination and using a nonvideo call.
**Relevance**
Our findings can support practitioners to adapt to telemedicine and understand the perspectives of their patients and other clinical colleagues.

Guidelines were published by the National Institute for Health and Care Excellence (NICE) in March 2020, which outlined recommendations for the delivery of cancer care in the United Kingdom during the COVID-19 pandemic.^6^ These included the postponing of nonessential procedures, prioritizing patients for systemic treatment, and conversion of face-to-face (FTF) appointments to telemedicine where appropriate. Where FTF appointments were required, it was advised that patients attend alone. As a result, this study aims to assess how the changing role of FTF consultations and the evolution of telemedicine affect professionals and patients across Scotland during the pandemic.

Telemedicine as a tool for the delivery of health care to populations with limited access to care has been increasing in usage since its conception in 1967.^7,8^ Available literature suggests that telemedicine is largely equivalent to in-person care and translates to high levels of satisfaction for both patients and care providers.^9^

There is a shortage of literature related to telemedicine in oncology,^10^ especially in the management of rare cancers such as sarcomas. Patients with rare cancers in Scotland are often required to travel significant distances to access care at one of the five cancer centers, three of which are major oncology centers found in Edinburgh, Aberdeen, and Glasgow. These large distances present both time and financial pressures to patients and can present barriers to care for some patients.

The aim of this study is to add to the increasing body of literature looking at the changing landscape of cancer care delivery in the United Kingdom,^11^ particularly looking at the use of telemedicine.

## METHODS

Patients were given telemedicine appointments with the consideration of the National Institute of Health and Care Excellence (NICE) guidelines^6^ and also relevant internal policies. The movement of patients from a traditional FTF appointment to telemedicine was ultimately at the discretion of the treating physician with consideration of the preferences of the patient. Patients who had progressive disease widely remained FTF along with those who required urgent assessment. Telemedical appointments were offered to patients in advance of regularly scheduled appointments.

Between June 8 and August 25, 2020, patients were invited to participate in an anonymous online survey questionnaire, with the option of completing a paper copy (Data Supplement), and professionals from the sarcoma multidisciplinary teams (MDTs) were also invited to complete a separate online survey questionnaire (Data Supplement). These surveys were modified versions of previously published surveys^11,12^ and probed participants for their views on how their provision of care had been changed by the evolving role of telemedicine, moving away from FTF appointments. Data were extracted, and descriptive statistics were calculated using SPSS. Some questions allowed the participant to expand on their thoughts, providing further depth to the analysis.

For the purpose of this study, telemedicine was defined as being any appointment, which was not undertaken FTF, for example, a telephone or video consultation.

## RESULTS

### Patient Survey on Telemedicine

#### Patient characteristics

A total of 74 patients participated with a median age of 55 years (range, 19-85 years; Table 1). All participants indicated their sex as either male or female in equal sample size (n = 37; Table 1). Patients were asked to self-identify their ethnicity, with 100% (n = 74; Table 1) of participants indicating that they were European or White. The majority of participants were educated and had received education at college level or above (n = 55; 74.3%; Table 1).

**TABLE 1:**
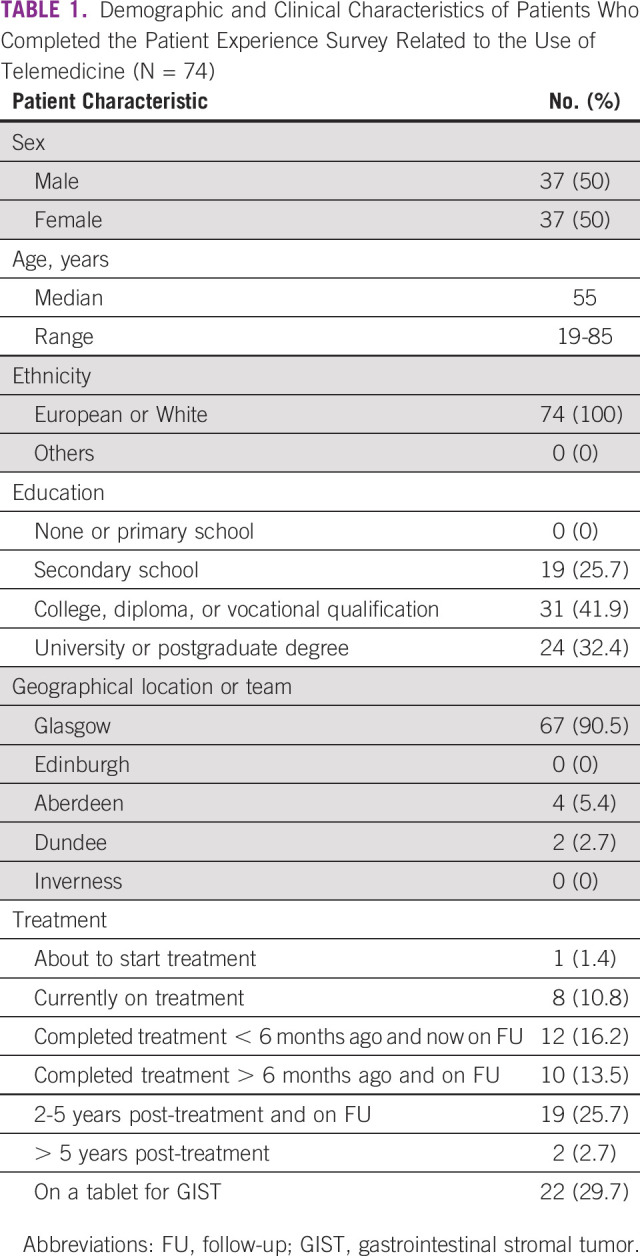
Demographic and Clinical Characteristics of Patients Who Completed the Patient Experience Survey Related to the Use of Telemedicine (N = 74)

#### Diagnosis and treatment

Most patients were being cared for by teams based in Glasgow (n = 67; 90.5%; Table 1), with the remaining participants being cared for by teams based in Aberdeen (n = 4; 5.4%) and Dundee (n = 2; 2.7%), and one respondent did not indicate. Participating patients most often finished treatment at the time of surveying (n = 43; 58.1%; Table 1).

#### Appointments and attitudes toward telemedicine

Patients were asked whether they had had an appointment regarding their care since March 24, 2020 (1 day after the initiation of lockdown in Scotland). The majority of patients had received FTF, telephone, or video consultation appointments (n = 74; 99%), with only one participant not having had an appointment in this time period (1%). Of those participants who had received an appointment, 54 patients had had one or more telephone appointments with a member of the sarcoma team (72%), 10 had had one or more FTF appointments (13%), nine had had an FTF appointment and then subsequently had telephone appointments (12%), and one had had another form of telemedicine appointment such as video consultation (1%).

Patients who had received FTF appointments reported high satisfaction with the consultation, with extremely satisfied being the most commonly reported score (mean = 9.59/10). Telemedicine appointments had similar satisfaction scores, with telephone and video appointments having mean satisfaction scores of 9.43/10 and 9.47/10, respectively. However, satisfaction scores were more variable with telephone appointment scores ranging from 2-10 of 10 and video from 5-10 of 10. FTF appointment scores ranged from 8 to 10 of 10. More than half of patients who had received a telemedicine appointment (by either telephone or video call) had met the person who performed their consultation previously (n = 32; 55.17%).

**FIG 1:**
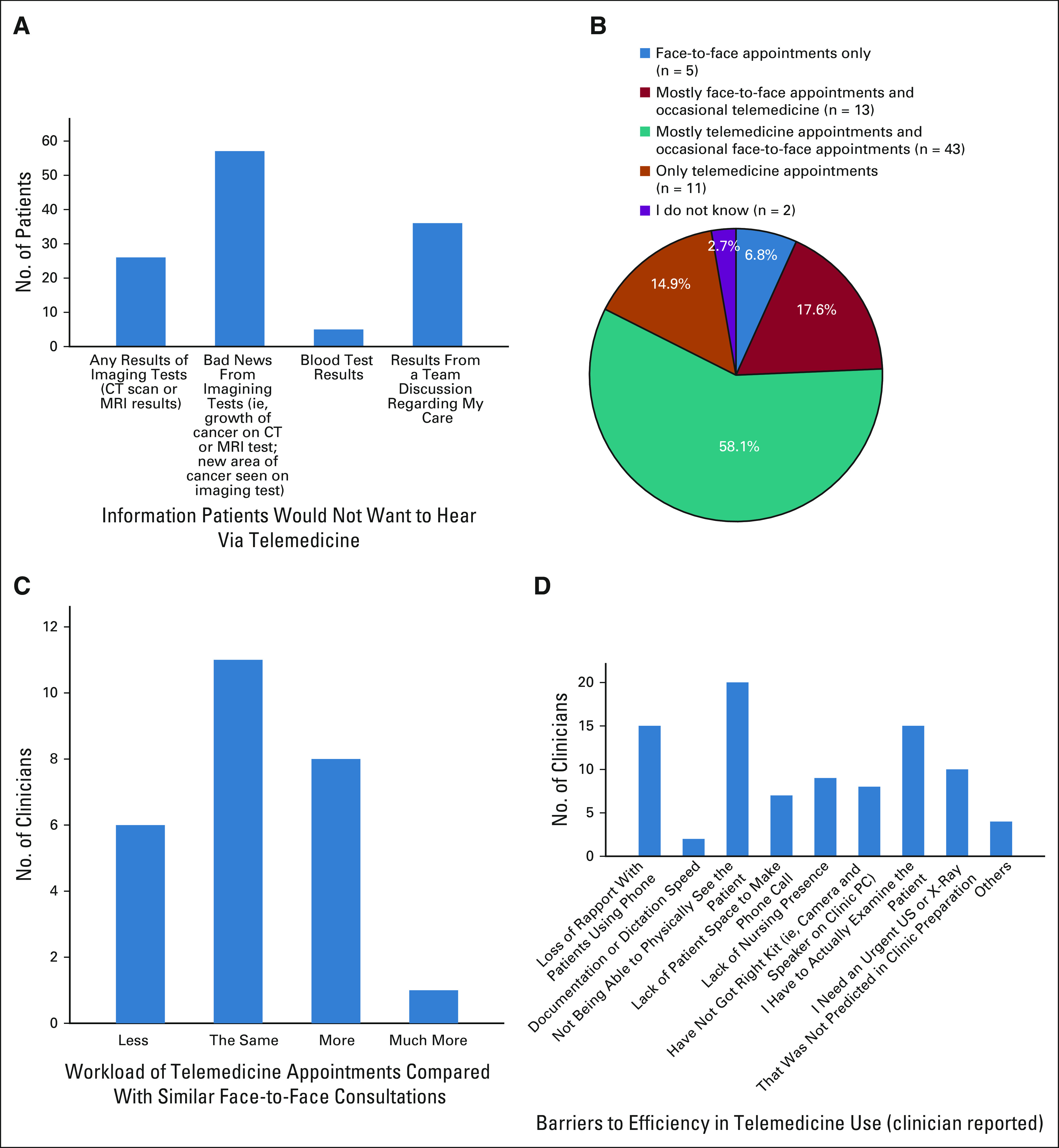
Summary of key patient and provider survey findings. (A) Information that patients would not want to hear via telemedicine. (B) Patient preferences for future modes of consultation. (C) Clinician-reported workload associated with telemedicine appointments when compared to similar face to face consultations. (D) Clinician-reported barriers to efficiency in telemedicine. CT, computed tomography; MRI, magnetic resonance imaging; PC, personal computer; US, ultrasound.

When asked going forward how they would like their appointments to be performed, patients indicated a preference to have mostly telemedicine with occasional FTF appointments (n = 43; 58.1%; Fig 1B). Commonly cited factors for this decision include reduced time traveling to hospital (n = 22), reduced cost to travel to hospital (n = 18), reduced time waiting in hospital (n = 24), and it being more convenient (n = 32). Patients also indicated the preference for only telemedicine appointments (n = 11; 14.9%). Patients who preferred mostly FTF appointments (n = 13; 17.6%) cited that they would find it more reassuring (n = 13). Patients currently being treated or had completed treatment in the last 6 months were more likely to indicate a preference for mostly or entirely FTF appointments (n = 10) than patients on follow-up treatment. Age, sex, and level of education in our sample did not affect mode of consultation preference.

The most commonly reported information that patients would not like to be told of via telemedicine was bad news from imagining results (eg, growth of cancer from magnetic resonance imaging scan or identification of new area of cancer; n = 57; 89.1%), with 40.6% not wanting to hear any scan results (n = 26). Patients also indicated that they would not like to have the results from discussions from their care team delivered via telemedicine (eg, need for referral to a surgeon to manage their cancer; n = 36; 56.3%). Few patients would not want to hear results of blood tests via telemedicine (n = 5; 7.8%; Fig 1A).

Patients reported that they would like to have a specialist nurse participate in telemedicine consultations on some occasions, such as when being told bad news (n = 33; 44.6%). Patients showed a preference for being able to request the attendance of a specialist nurse (n = 33; 44.6%). A large proportion of patients would like to always have a specialist nurse present (n = 26; 35.1%).

### Provider Survey on Telemedicine

#### Provider characteristics

Most providers who were invited to participate responded to the provider survey (N = 26). Most providers were physicians (eight consultant surgeons, six consultant oncologists, and five registrars [training oncologists]), with the remaining respondents being nurses (six clinical nurse specialists and one research nurse); most had worked on the sarcoma unit for > 5 years (n = 13; 50%) or < 2 years (n = 8; 31%; Table 2). Most were based in the West of Scotland (n = 12; 46%) with the remaining being based in the North of Scotland (n = 8; 31%) and the South East of Scotland (n = 6; 23%).

**TABLE 2:**
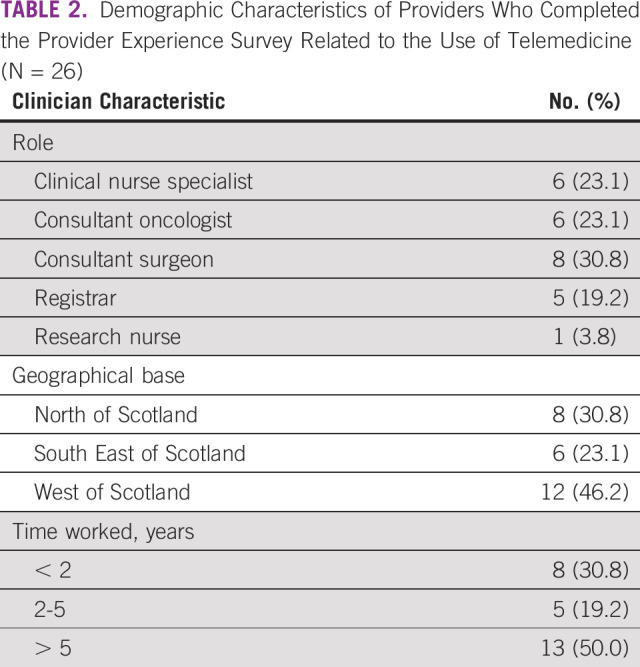
Demographic Characteristics of Providers Who Completed the Provider Experience Survey Related to the Use of Telemedicine (N = 26)

#### Provider attitudes toward telemedicine

The majority of providers reported that telemedicine appointments took the same amount of time compared with FTF appointments (n = 12; 46%). Providers reported that lack of physical examination in telemedicine appointments sometimes negatively affected their ability to provide care (n = 12; 46%). Most reported that the use of telemedicine did not increase their workload (n = 17; 65%), with the majority indicating that workload was the same as FTF appointments (n = 11; 42.3%; Fig 1C).

**FIG 2:**
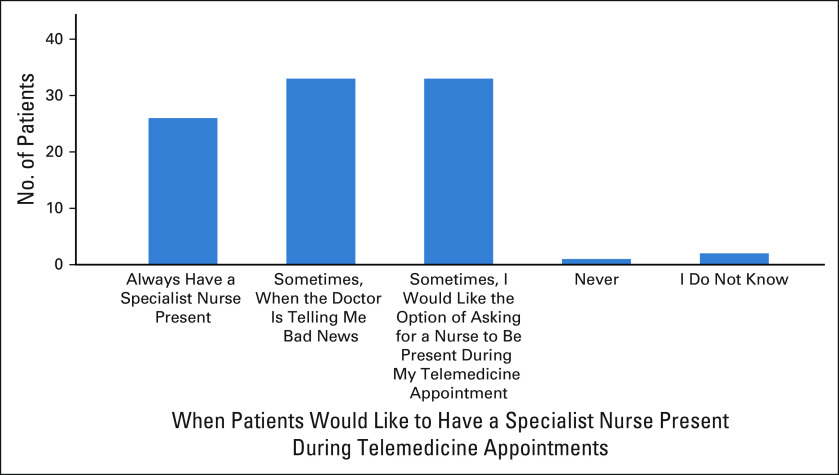
Patient preferences for occasions to have a specialist nurse present during telemedicine consultations.

Most commonly reported barriers to efficiency when using telemedicine were not being able to physically see the patient if using a nonvideo call (n = 20; 77%), lack of ability to undertake physical examination (n = 15; 58%), and loss of rapport (n = 15; 58%; Fig 1D). The majority of providers indicated that their experience with telemedicine would be improved by the use of video-enabled telemedicine as opposed to a telephone call (n = 20; 80%) and better infrastructure (eg, private office, headset, etc; n = 11; 44%; Fig 2).

Providers most commonly indicated that telemedicine should become part of regular practice (n = 17; 66%); follow-up appointments for patients on surveillance were indicated as suitable (n = 24; 96%) along with follow-up appointments for patients on stable doses of oral anticancer treatments (n = 14; 56%). Only three respondents reported that telemedicine should not be implemented into practice postpandemic and patients should always be seen FTF as before, all of whom were Clinical Nurse Specialists. Most preferred having time during a specific existing in-person clinic for telemedicine (n = 17; 65%).

Most clinicians or MDT members did not indicate a preference for specialist nurses to be present during telemedicine consultations (n = 16; 61.5%); Consultant Oncologists and Registrars are more likely to indicate a preference for their presence (n = 8; 72.7%). Negative impacts on education of trainees were of moderate to high concern with 18 providers scoring a negative impact > 5 of 10 (72%).

## DISCUSSION

Telemedicine in the setting of this study was implemented under nationwide NICE guidance and so did not come about naturally. Uptake was required in a time-pressured environment presenting a steep learning curve for both patients with sarcoma and sarcoma providers. Data extracted from our research study indicate positive results regarding patient and provider satisfaction with telemedicine in this setting.

Clinician workload was reported to be largely the same when using telemedicine when compared with FTF appointments. Patients reported to have similar satisfaction with telemedicine appointments when compared with those who received FTF appointments, although satisfaction with the former was more variable. This should be the focus of future work to investigate and mitigate the root causes of dissatisfaction in patients related to telemedicine consultations.

There was a general consensus with providers that telemedicine should play a role in the delivery of care to patients with sarcoma postpandemic for certain populations, in particular, follow-up surveillance appointments and for those who were on stable doses of oral anticancer medications. Findings by Smrke et al (2020) were congruent, with 89% of clinicians in their sample indicating these populations as appropriate for telemedicine. Patients echoed this feeling, preferring to have predominantly telemedicine appointments with occasional FTF appointments. Patients were receptive to receiving telemedicine consultations, given that they were reassured that a physical examination was not needed. This indicates that going forward, telemedicine should be used for certain patient populations.

Perhaps unsurprisingly, the majority of patients were opposed to receiving bad news from imaging results via telemedicine. It is a common phenomenon that patients are opposed to hearing bad news via telemedicine.^10,13^ This is in contrast to another study of patients with sarcoma during the pandemic, in which a large proportion of patients were not opposed to hearing bad news via telemedicine.^11^ This discrepancy requires exploration in future studies.

Providers indicated that telemedicine should be integrated into routine provision of care in patients with sarcoma, citing that video-enabled telemedicine was an important improvement, which should be made along with improvements in infrastructure. The Scottish National Health Service during the pandemic has made concerted efforts to improve their information technology infrastructure^14^ with the adoption of video-enabled call platforms such as Near Me and Microsoft Teams.

Certain providers felt that the presence of a specialist nurse during the clinical encounter would be an important addition, with patients echoing this sentiment. We should emphasize the importance of stakeholder confidence in the use of telemedicine; the care of patients with sarcoma in Scotland involves not only multiple stakeholders across systems including but not limited to the MDT teams in hospital settings, but also pharmacy workers and care staff externally, as noted by Smrke et al.^11^ In particular, pharmacy teams are essential in the provision of treatments for patients with gastrointestinal stromal tumors who had supplies sent to their home addresses during the pandemic.

Future work should be carried out to assess the impact of telemedicine on patient outcomes as we move into a new care delivery model. Our sample lacked representation from ethnic minorities and so future work should be complete to include this population, in particular, the role of the interpreter should be explored.

In conclusion, this study provides valuable insights into the benefits of telemedicine in the provision of care for patients with rare cancers. Although the mode of implementation was required given a global health emergency, there has been increasing interest over the past couple of decades on how the use of telemedicine can alleviate pressures on the health system and increase access to specialist care for patients with rare cancers. This should be seen as an opportunity to redesign the provision of cancer care in Scotland for the benefit of the patient, the provider, and the wider health system.

The experience of both patients and providers was broadly positive. Patients preferred to have bad news delivered FTF. Given that providers largely reported similar workloads associated with telemedicine compared with FTF appointments and patients had fewer time and cost pressures, it is recommended that this mode of care delivery be implemented into routine care.
